# Gas Selectivity Enhancement Using Serpentine Microchannel Shaped with Optimum Dimensions in Microfluidic-Based Gas Sensor

**DOI:** 10.3390/mi13091504

**Published:** 2022-09-10

**Authors:** Maryam Aghaseyedi, Alireza Salehi, Shayan Valijam, Mostafa Shooshtari

**Affiliations:** 1 Department of Electrical Engineering, K.N. Toosi University of Technology, Tehran 1631714191, Iran; 2 Laboratory of Electronic Components, Technology and Materials (ECTM), Department of Microelectronics, Delft University of Technology, 2628 CD Delft, The Netherlands

**Keywords:** microfluidic, microchannel, gas sensor, gas response, selectivity

## Abstract

A microfluidic-based gas sensor was chosen as an alternative method to gas chromatography and mass spectroscopy systems because of its small size, high accuracy, low cost, etc. Generally, there are some parameters, such as microchannel geometry, that affect the gas response and selectivity of the microfluidic-based gas sensors. In this study, we simulated and compared 3D numerical models in both simple and serpentine forms using COMSOL Multiphysics 5.6 to investigate the effects of microchannel geometry on the performance of microfluidic-based gas sensors using multiphysics modeling of diffusion, surface adsorption/desorption and surface reactions. These investigations showed the simple channel has about 50% more response but less selectivity than the serpentine channel. In addition, we showed that increasing the length of the channel and decreasing its height improves the selectivity of the microfluidic-based gas sensor. According to the simulated models, a serpentine microchannel with the dimensions W = 3 mm, H = 80 µm and L = 22.5 mm is the optimal geometry with high selectivity and gas response. Further, for fabrication feasibility, a polydimethylsiloxane serpentine microfluidic channel was fabricated by a 3D printing mold and tested according to the simulation results.

## 1. Introduction

Presently, there is a growing demand for microfluidic-based gas-sensing techniques rather than traditional methods such as gas chromatography (GC) and mass spectroscopy (MS) [1,2,3,4]. A microfluidic-based gas sensor is generally composed of a single sensor at the end of a microchannel to detect different analytes [5]. The microfluidic-based gas sensor has significant merits, such as small size, high accuracy, low cost and low power consumption compared to traditional methods, that cause the microsystem to be used in a wide range of applications, such as environmental monitoring, biochemical analysis, safety, food quality control, etc. [2,6]. Moreover, the microfluidic channel provides a specific route for the gas flow and can control ambient gas parameters such as volume, flow rate and temperature [7,8,9]. Several parameters affect the gas-sensing mechanism of microfluidic-based gas sensors such as microchannel geometric design [1,10] and channel material [11]. The optimal channel design by changing the length, height and shape has a great impact on the performance of the gas sensor [1,12].

Microfluidic channel material also affects the gas-sensing parameters. Polydimethylsiloxane (PDMS), as a nonpolar material, is a common matter for microfluidic channel fabrication due to its high hydrophobicity. PDMS has considerable merits in comparison with other materials, especially polymeric ones such as poly (methyl methacrylate) (PMMA) and poly carbonate (PC) [11]. In addition, PDMS is elastic, transparent and nontoxic [11,13]. Any sophisticated channel design can be easily formed in PDMS using soft lithography techniques such as rapid prototyping and replica molding rather than the etching method [14]. Nonpolar PDMS tends to absorb nonpolar analytes rather than polar ones [15,16]. It is believed that polar analytes have higher diffusion coefficients and less interaction with channel walls than nonpolar analytes [17]. However, among polar analytes, the adsorption probability of the analytes with lower diffusion coefficients to the PDMS channel walls is higher than for other polar analytes, and it increases by improving the channel geometry. In fact, by increasing the length and serpentine shape of the PDMS channel and decreasing its height, the adsorption probability of polar analytes to the channel walls increases [11].

Moreover, PDMS can be used not only as a channel material but also as a sensing layer for volatile organic compounds (VOCs). Nonpolar PDMS swells when it adsorbs nonpolar VOC molecules, and its swelling varies as a function of VOC concentration [18,19]. VOCs, as a family of carbon-containing chemicals, have exhibited high vapor pressure at room temperature [20,21], and they are the most important source of indoor environmental pollutants [22,23,24]. Therefore, detecting, monitoring and discriminating these vapors is imperative.

There are several reports of simulating sensors based on microfluidic channels [25,26]. Simulation using software is the basis for the fabrication of microfluidic sensors. Simulation using COMSOL software is one of the best ways because it can solve coupled multiphysics phenomena simultaneously and provides a unified workflow for electrical, mechanical, fluid, acoustics and chemical applications. However, studying the shape of the channel with an emphasis on selectivity and comparison with experimental results has also received less attention.

In this study, a simulation of a microfluidic-based gas sensor is presented and compared in both serpentine and simple models. An optimal microchannel is designed by considering the effects of geometric parameters to improve gas-sensing results. Here, PDMS is considered as the channel material due to its high hydrophobicity. Then, the impacts on analyte adsorption to channel walls are investigated due to channel shape, considering the modeling of diffusion, surface adsorption/desorption and surface reactions. In the end, the simulation results are verified/validated against the data obtained from experimental tests.

## 2. Theory

**Figure 1:**
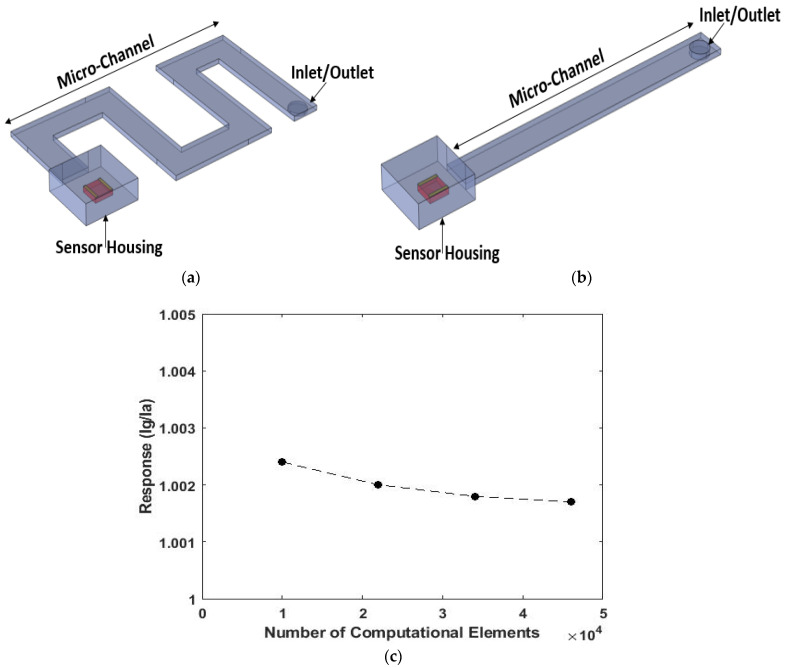
Schematics of the (**a**) serpentine and (**b**) simple simulations. In each model, the gas inlet/outlet, microchannel and sensor housing are specified. (**c**) Result of the mesh independency analysis.

Three-dimensional models of a microfluidic-based gas sensor were simulated using COMSOL Multiphysics 5.6 (COMSOL Inc, Stockholm, Sweden). Designed schematics of serpentine and simple microchannels in COMSOL software are shown in Figure 1. The sensor housing and gas inlet/outlet are marked in these two different microchannel structures. These two models were designed according to three principles: (1) mass diffusion, (2) the physical adsorption and desorption of the gas molecules to/from the channel walls and (3) surface reactions [1,10]. Moreover, a model user-controlled mesh was applied to solve defined 3D models for the microsystem using free tetrahedral meshes of different sizes. The mesh sizes depended on the dimensions of the microsystem. The mesh size of the microchannel and sensor housing was defined using the normal mode (maximum element size = 2.85 mm and minimum element size = 0.51 mm). In addition, the mesh size of the sensor was defined using the fine mode (maximum element size = 2.28 mm and minimum element size = 0.28 mm). The response of the sensor at the end of the channel with the reference dimensions of W = 3 mm, H = 80 µm and L = 30 mm is depicted versus the number of computational elements in Figure 1c. By increasing the number of elements, the sensor response decreased. However, the value of the responses at a different number of elements made very little difference. Therefore, we considered the total number of elements in the mesh structure to be 10,000. In each computational step, the channel inlet was exposed to a specific concentration of analyte (acetone and ethanol) at t = 0 s, and then it was recovered in fresh air at t = 40 s. The theories of three phenomena and their effects on the sensing response and selectivity are investigated in the following subsections.

### 2.1. Diffusion Model

The concentration gradient inside and outside the microfluidic channel leads to the diffusion process. By applying a certain gas concentration to the channel inlet, the gas diffuses inside the channel during the gas exposure time. On the other hand, after cutting it off, the gas will diffuse out of the channel from the outlet during the cutting time [27]. The Maxwell–Stefan mass transfer equations provide an acceptable approximation of multicomponent diffusion in gases at a low density. When a specified analyte diffuses in a homogeneous compound, the Maxwell–Stefan multicomponent mass transfer equation is applied to describe the relationship between the diffusion process and the gradient concentration in a microfluidic channel according to Equation (1) [28,29]:

(1) $$\nabla x_{i}=-\overset{n}{\underset{j=1}{\sum }}\frac{x_{i}x_{j}}{C_{\mathrm{tot}}D_{\mathrm{ij}}}\left(J_{j}-J_{i}\right)$$

where x_i_ and x_j_ are the mole fractions of components I and j, respectively; $J_{I}$ and are the velocity of component I and j, respectively; D_ij_ refers to the binary diffusion coefficient of component I in component j; C_tot_ is the mixture concentration and n represents the number of a component system. Diffusion coefficients specify the quantity of a gas that, in diffusing from one area to another, passes through each unit of cross section per unit of time when the volume–concentration gradient is in unity [30]. The diffusion coefficient values were calculated using the parameters obtained from the literature. Acetone and ethanol are two reductive gases with diffusion coefficients of $10.49\times 10^{-2}\frac{{\mathrm{cm}}^{2}}{s}$ and $11\times 10^{-2}\frac{{\mathrm{cm}}^{2}}{s}$, respectively, which are examined in this research [30,31].

### 2.2. Adsorption and Desorption Model

Gas molecules adsorb/desorb on the surfaces of microchannel walls. According to the Langmuir adsorption isotherm, an adsorbate molecule reacts with the surface according to the following chemical reaction [1,32]:

(2) $$A_{g}+S⇆{\;A}_{\mathrm{ad}}$$

where A_g_ is adsorbate gaseous molecules, S is vacant sites on the surface and $A_{\mathrm{ad}}$ is adsorbed species. The rate of adsorption/desorption can be written using the first-order reaction as follows [33,34]:

(3) $$\mathrm{Rate}\;\mathrm{of}\;\mathrm{adsorption}/\mathrm{desorption}\;(R_{\mathrm{ad}})=K_{L}\frac{\left[C_{A_{g}}\right][C_{S}]}{\left[C_{A_{\mathrm{ad}}}\right]}$$

where $\left[C_{A_{g}}\right],$ $[C_{S}]\;\mathrm{and}\;[C_{A_{\mathrm{ad}}}]$ represent the concentrations of adsorbate gas molecules, vacant sites on the surface and adsorbed species, respectively, and $K_{L}=\frac{k_{A}}{k_{D}}$ refers to the Langmuir constant, as k_A_ and k_D_ are the adsorption and desorption reaction constants.

K_A_ and k_D_ are defined as Equations (4) and (5), respectively [35]:

(4) $$k_{A}=\frac{\sigma A}{\sqrt{2\pi {\mathrm{mk}}_{B}T}}$$

where A is the area of one vacant site or adsorbed gas molecule, σ refers to the absorption probability of the surface, $k_{B}$ is the Boltzmann constant and m and T refer to mass and temperature of the surface, respectively.

(5) $$K_{D}=\frac{1}{\tau }\;,\;\tau =\tau_{0}\exp\left(\frac{Q}{R_{g}T}\right)$$

where Q, $\tau_{0}$ and R_g_ are the heat of absorption, the residence time constant and universal gas constant, respectively.

As shown in Equations (4) and (5), k_A_ and k_D_ depend just on the channel surface and change by varying it.

### 2.3. Gas Response Model

A common n-type metal oxide semiconductor (MOS) sensor (ZnO) with electrodes made of gold was defined to investigate the effect of the channel on the response of the sensor system. The ZnO sensor with dimensions of 2 mm×2 mm×0.5 mm and two stripe electrodes with dimensions of 0.2 mm×2 mm×0.1 mm was placed in the position sensor housing in Figure 1. Although most characteristics of ZnO and Gold are defined in the COMSOL Multiphysics software by default, some other essential properties, such as electrical conductivity, relative permittivity, etc., must be added to the model according to the working temperature and morphology of ZnO to observe its reactions with the target gases. In addition, all properties of the sensitive layer were set to be constant for all experiments. Moreover, a dc voltage was applied to the electrodes by defining the electric current (EC) physics for them, and a time-dependent study was defined to solve the gas response model. Due to the potential difference between the electrodes, there were different current variations on the sensitive layer when it was in contact with the target gas and fresh air, which showed the sensing and basic responses, respectively.

The gas response of the sensor is calculated based on current variation according to Equation (6):

(6) $$\mathrm{Response}=\frac{I_{g}}{I_{a}}$$

where $I_{g}\;$ and $I_{a}$ are the current of the sensor in contact with the target gas (sensing response) and fresh air (basic response), respectively.

The exposure of the microfluidic-based gas sensor to different analytes at the same concentration leads to different responses due to the sensor reactions [10]. In an n-type ZnO sensor, oxygen molecules are adsorbed on the ZnO surface and form oxygen ion molecules by attracting an electron from the conduction band of a sensor, as shown in Equation (7).

(7) $$O_{2}\left(\mathrm{gas}\right)+{\;e}^{-}↔{\;O}_{2}{}^{-}\;\left(\mathrm{adsorb}\right)\;\;\;\;$$

The oxygen ions on the ZnO surface are active with the gas molecules and give the electrons from the surface back to the conduction band of ZnO. Therefore, the conductivity of the sensor changes due to the transfer of electrons between the analyte and the oxygen on the surface of the sensing layer (ZnO surface). A chemical reaction between gas molecules and oxygen ions on the sensing layer can be considered according to Equation (8):

(8) $$X+O_{\mathrm{ads}}{}^{b-}\to X^{\prime }+{\mathrm{be}}^{-}$$

where X and $X^{\prime }$ are the target gas and out gas, respectively, and the b value is the number of electrons. To show the sensor reactions in the simulation study, as mentioned before, electric current (EC) physics is used to plot the I–V or resistance curves of the sensing layer over time.

### 2.4. Selectivity Model

To compare the currents obtained from different analytes regardless of their concentrations and baseline variations, current normalization was used. The following formula (Equation (9)) was used to normalize the current in the simulation:

(9) $$I_{n}\left(t\right)=\frac{I\left(t\right)-\min\left(I\left(t\right)\right)}{\max\left(I\left(t\right)\right)-\min\left(I\left(t\right)\right)}$$

where $I_{n}\left(t\right)$, min (I(t)) and max (I(t)) are the normalized, minimum and maximum current at all simulation times, respectively. Selectivity is the ability to identify and distinguish gas A from gas B; it is represented by the ratio of the sensor signals induced by gas A and gas B [36,37]. The selectivity of a microfluidic-based gas sensor can be determined based on the difference between the normalized currents of two gases, as in the following equation:

(10) $$S_{\mathrm{AB}}=\overset{200}{\underset{t=0}{\sum }}\left|I_{\mathrm{nA}}\left(t\right)-I_{\mathrm{nB}}\left(t\right)\right|$$

where $S_{\mathrm{AB}}$, $I_{\mathrm{nA}}\left(t\right)$ and $I_{\mathrm{nB}}\left(t\right)$ are the selectivity indicator, the normalized current of gas A and the normalized current of gas B, respectively. $S_{\mathrm{AB}}$ represents the difference between $I_{\mathrm{nA}}\left(t\right)$ and $I_{\mathrm{nB}}\left(t\right)$, so the larger $S_{\mathrm{AB}}$ is, the greater the difference between gas A and gas B, and consequently they are more selectable.

## 3. Experimental Method

To obtain an accurate validation of the simulation results, a sample of the defined gas-sensing system was fabricated. The fabrication of the main gas-sensing system was divided into two parts: the growth of ZnO as a sensitive layer and the fabrication of the microfluidic PDMS channel. Moreover, VOC vapor sensing test methods were used according to references [20,22,38].

### 3.1. Microchannel Fabrication

The microfluidic channel design was created by COMSOL Multiphysics software and converted to “.stl” format, which is readable for most 3D printers, which have been recently used to fabricate microfluidic devices. There are different 3D printing technologies, including stereo lithography (SLA), poly jet, digital light processing, etc. [39]. They are classified based on resolution, speed and material. In this study, the mold of the microfluidic channel was fabricated with a high-speed SLA printer (Kavosh laser, Model: kavosh LCD, Tehran, Iran). Then, it was used to build the microchannel on PDMS, which was a commercial Sylgard 184 (Dow Corning, Midland, Michigan, United States) type. First, a 10:1 mixture of PDMS (liquid) and a cross-linking agent was prepared, poured into the mold and heated at 90 °C for about 2 h in the oven to obtain an elastomeric microchannel replica. Then, it was carefully peeled off the mold. To bond the PDMS layers, both the microchannel and cover layers were treated with plasma for about one minute at 20 mA and 65 mTorr then immediately brought into contact with each other. The bonded layers were heated at 90 °C for about one hour to strengthen the bonding. The details of the method for channel fabrication are described in Ref. [40].

**Figure 2:**
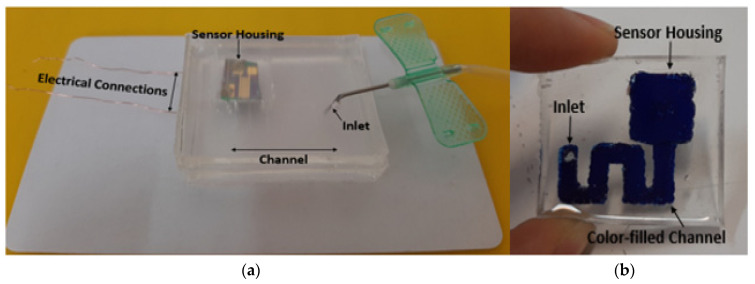
(**a**) The fabricated microfluidic-based gas-sensing system. (**b**) Colored channel of gas-sensing system for clarification.

The fabricated experimental gas-sensing system with a microfluidic channel is presented in Figure 2. The sensor housing, channel path and gas inlet location in the system are shown in Figure 2a. Figure 2b shows the channel, which is filled with blue color to clarify.

### 3.2. ZnO Growth

ZnO thin films were synthesized using the sol–gel method on a silicon substrate. First, a ZnO solution (1 M) was obtained by mixing 2.75 g of zinc acetate dehydrate (ZAD) as a precursor with 12.5 mL of isopropanol (IPA) as a solvent. The mixed solution was stirred on a hotplate at 60 °C for about 20 min to obtain a homogenous solution. Then, 1.5 mL of diethanolamine (DEA) was added dropwise to the ZnO solution to stabilize it while stirring constantly at 60 °C for 2 h. The solution was then stored for 24 h to age at room temperature before deposition. All materials used for zinc oxide growth were supplied by Merck company (Darmstadt, Germany).

**Figure 3:**
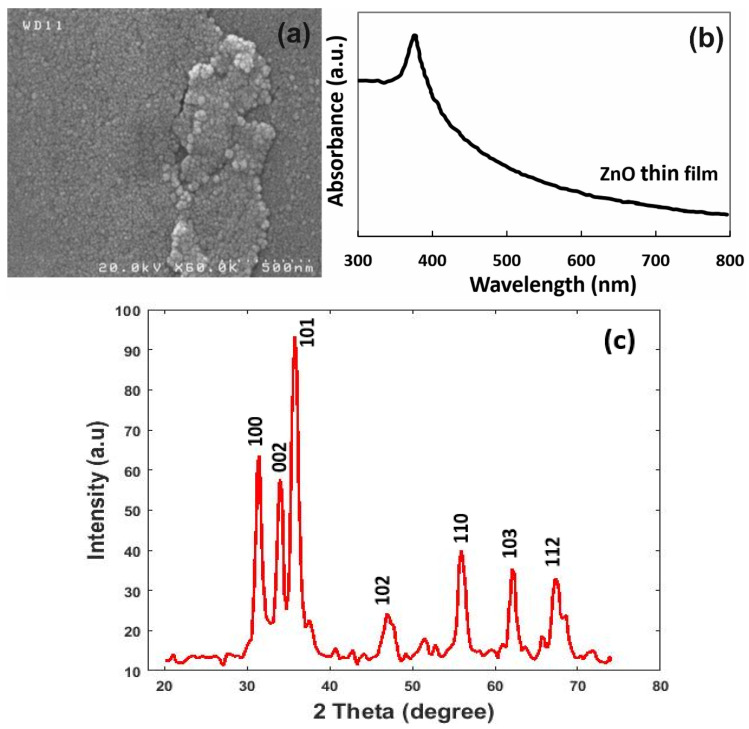
(**a**) SEM of ZnO thin film on the silicon substrate. (**b**) UV-Vis absorption spectrum of ZnO film. (**c**) XRD spectrum of ZnO thin film.

The morphology and microstructure of the ZnO thin film were examined by scanning electron microscope (SEM, Hitachi, Bridge Tronic Global, S4160, Costa Mesa, CA, USA). As shown in Figure 3a, the film exhibited a pore structure, which is important for the gas-sensing system. It can be estimated from the SEM image in Figure 3a that the grain size was less than 50 nm. To ensure the correct growth of ZnO, UV-visible spectroscopy (UV-Vis) was applied (Perkin Elmer, Lambda EZ201, Waltham, MA, US) [41]. Figure 3b illustrates the absorbance spectrum of ZnO thin films. It is clear from Figure 3b that strong absorption occurs in UV wavelength at 360 nm. A weak absorption area occurs for almost the entire visible field, ranging between 400 and 800 nm.

Moreover, to prove the presence of ZnO in the obtained sensor, X-ray diffraction spectroscopy (XRD, Thermo Scientific, ARL EQUINOX 3000, Waltham, MA, US) was employed [42]. The XRD patterns of the as-prepared sample are presented in Figure 3c. In the XRD patterns of the ZnO, the location and intensity of the diffraction peaks that were present at 31.48°, 33.99°, 35.79°, 46.91°, 55.93°, 62.08° and 67.27° can be indexed into the (100), (002), (101), (102), (110), (103) and (112) crystal planes of wurtzite-type hexagonal ZnO (JCPDS No. 65-3411) [43].

## 4. Results and Discussion

**Figure 4:**
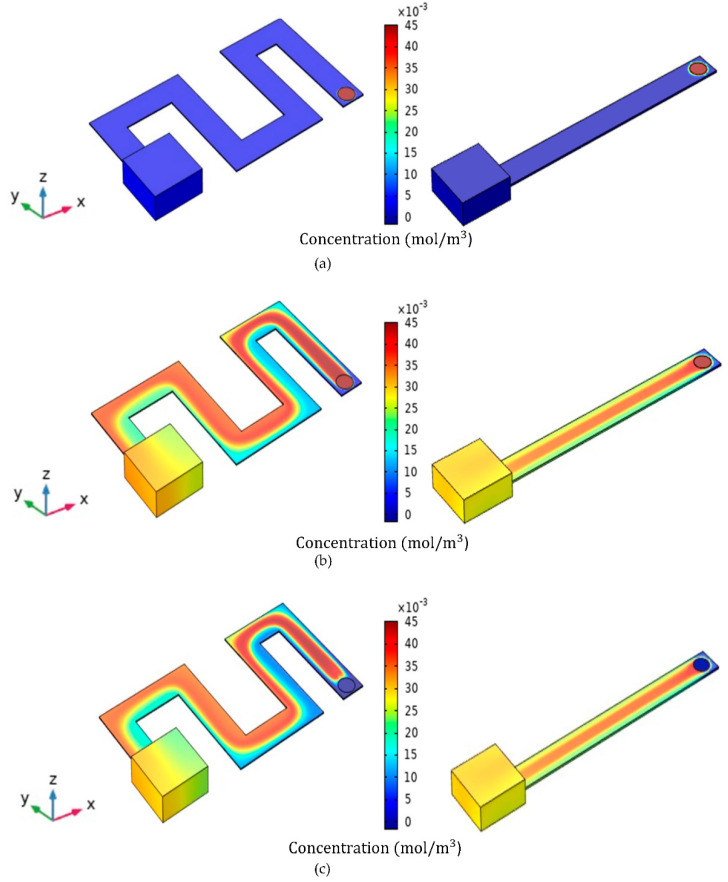

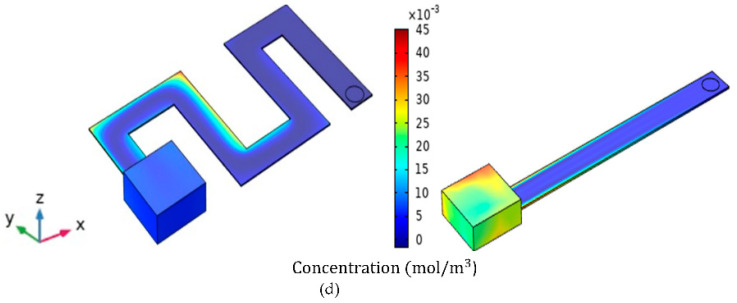
Visualization of the concentration profiles of the serpentine and simple models at the gas exposure and cutting times. (**a**) *t* = 0 s, (**b**) *t* = 25 s, (**c**) *t* = 40 s, (**d**) *t* = 100 s.

This paper investigated the effects of some parameters on the microfluidic-based gas sensor to design an optimal microchannel. The two models introduced in Figure 1 were defined with dimensions of W = 3 mm, H = 80 µm and L = 30 mm. According to the simulated model shown in Figure 4, at t = 0 s (Figure 4a), the inlet of the channel is exposed to the target gas with a certain concentration. At t = 25 s (Figure 4b), gas molecules diffuse along the channels, leading to a concentration gradient and an increase in the volume of gas molecules reaching the sensor housing. At t = 40 s (Figure 4c), the concentration of the target gas at the inlet point equals zero. In other words, fresh air diffuses into the channels at t = 40 s. From this time, the gas molecules inside the channels flow to the outlet, and the gas concentration inside the sensor housing decreases gradually (Figure 4d). All simulations were carried out at room temperature (27 °C) and normal air pressure ($1.013\times 10^{5}\;\mathrm{pa}$), and the relative humidity remained at 20%. Moreover, a 2.6 ppm concentration of acetone gas was exposed to the channel inlet, and its effects on the response and selectivity were compared in the simple and serpentine models.

**Figure 5:**
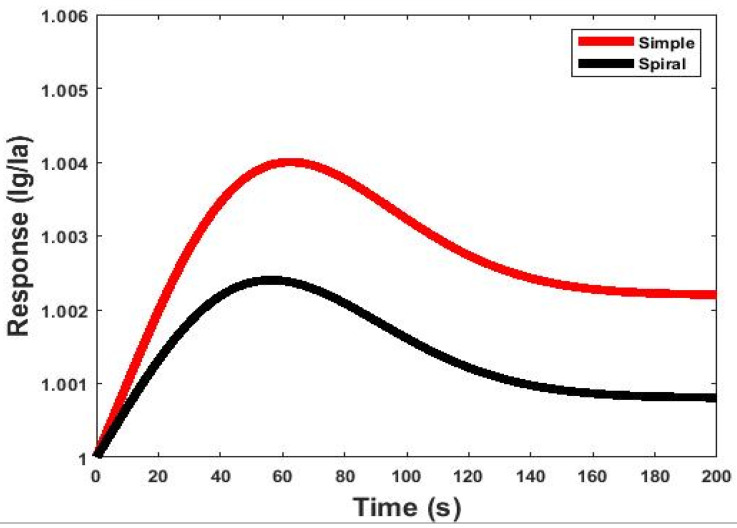
The gas response of the simple and serpentine models of microfluidic-based gas sensors with the same length and height at 2.6 ppm concentrations of acetone gas operated at room temperature.

By obtaining currents from simulation, the responses to gas for both sensors were calculated according to Equation (6). The results of simulating the responses of the gas sensors for 200 s are shown in Figure 5. Figure 5 shows the responses of the sensors exposed to 2.6 ppm at a 27 °C working temperature. The response of the gas sensor at the end of the serpentine microchannel was lower than the simple model. This issue refers to the high adsorption probability of the analytes to the serpentine channel walls compared to the simple model. In fact, as the channel becomes more serpentine, the collision of gas molecules with each other and the channel walls increases, and their diffusion rates inside the channel decrease. Therefore, most of the molecules tended to adsorb on the channel walls, and fewer gas molecules diffused toward the sensor, leading to a decrease in the response. As Figure 5 shows, the response of the simple channel model was about twice as sensitive as the response of the serpentine channel model with the same dimensions. However, diffusion was the main parameter in the simple model affecting the sensor’s response at the end of the microchannel, and more gas molecules reached the sensor.

According to recent results, diffusion and adsorption mechanisms are effective parameters on the gas response that act differently in each of the two model channels with the same dimensions. Since dimensional channel variation affects the adsorption/desorption and diffusion mechanisms, these changes can also affect the gas response.

In this section, the simulated model was used to study the effects of different dimensions, such as the length, height and shape of the two model channels, on the response and selectivity between two different gases (acetone and ethanol). In different situations, the length and height of the channel were changed in comparison with the reference dimensions (W = 3 mm, H = 80 µm, L = 30 mm). Here, nine different dimensions were considered to determine the optimal geometry for reaching the maximum response and selectivity, as presented in Table 1. In each of the simulation rounds, one dimension changed and the other dimensions remained fixed. The simulation results were investigated and compared for each round.

**Figure 6:**
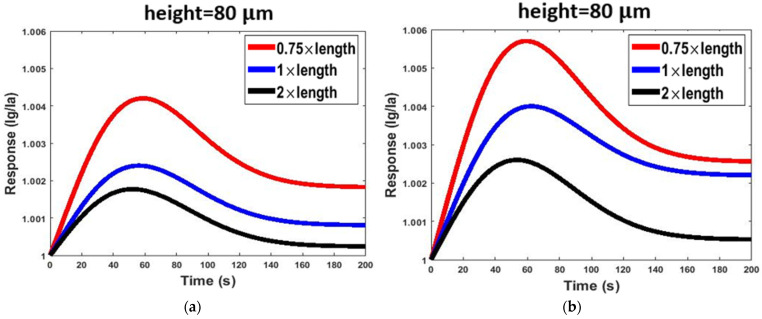
The effect of length variation of the channel on the responses of two sensor structures at 2.6 ppm concentrations of acetone gas operated at room temperature: (**a**) serpentine model and (**b**) simple model.

In the simple model, only the length and cross-section of the channel affected the diffusion and adsorption processes. However, the shape of the channel was also an effective parameter on the diffusion and adsorption processes in the serpentine model. Figure 6 shows the effect of channel length on the gas response of the two sensor structures. Generally, if the length of the channel (simple and serpentine models) increased, the diffusion rates of gas molecules inside the channel decreased. This deceleration is due to the continual collision of gas molecules with each other and channel walls [44]. Therefore, it takes a long time for gas molecules to reach the sensor housing. In addition, it is also conceivable that increasing the collisions along the channel path leads to the loss of kinetic energy of a large number of gas molecules. These gas molecules do not reach the sensor housing and, as a result, are ineffective in the response of the sensor. It can be concluded that increasing the channel length leads to a decrease in gas concentration in the sensor housing. Moreover, the PDMS can adsorb VOC molecules [19]. Therefore, by increasing the channel length, more adsorption sites are exposed to gas molecules. The adsorption of VOC molecules by the PDMS, again, leads to a decrease in the gas concentration in the sensor housing. As shown in Figure 6, increasing the length from 22.5 to 60 mm led to a 56% reduction in the response in the serpentine model and a 54% reduction in the response in the simple model.

**Figure 7:**
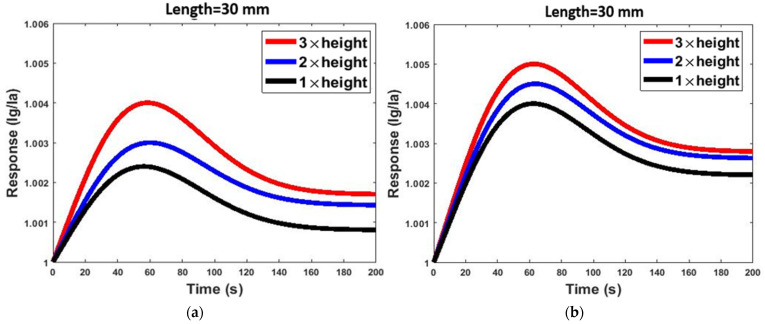
The effect of height variation of the channel on the response of two sensor structures at 2.6 ppm concentrations of acetone gas operated at room temperature: (**a**) serpentine model and (**b**) simple model.

As Figure 7 shows, channel cross-section changes affect the responses the same as the length variation. In this study, we modeled cross-sectional changes with channel height changes, and we kept the channel width constant (see Table 1). Decreasing the channel height slowed down the diffusion and transfer rates of molecules and increased the effectiveness of physical adsorption [3], and thus the response of the sensor decreased. By increasing the length, the reduction in the cross-sectional area increased the collision of gas molecules with each other and the channel walls. As shown in Figure 7, increasing the cross-sectional area from 80 to 240 μm led to a 67% increase in the response in the serpentine model and a 25% increase in the response in the simple model.

**Figure 8:**
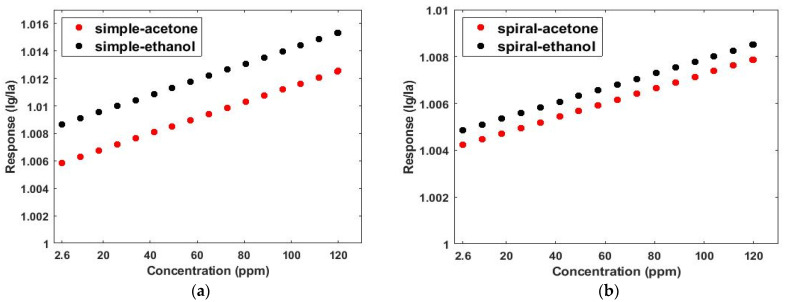
The relationship between the gas response of each model and the concentration of acetone and ethanol at room temperature: (**a**) simple model and (**b**) serpentine model.

According to the obtained simulation results, the microchannel with dimensions of W = 3 mm, L = 22.5 mm and H = 240 mm had the highest and most optimal response among the different geometries. However, the serpentine channel structure reduces the concentration of gases reaching the sensor housing and is less sensitive. Generally, gas molecules diffuse along the simple channel easier and faster than the serpentine channel to reach the sensor, and it is obvious that if the gas concentration increases, more gas molecules diffuse toward the sensor. In addition, the relationships between the response and the concentrations of acetone and ethanol were investigated. Figure 8 shows the effect of concentration changes (from 2.6 ppm to 120 ppm) of acetone and ethanol gases on the responses of two sensor structures with the same dimensions operated at room temperature. The responses of the two models increased linearly as the concentration of each target analyte increased. This phenomenon occurred because more gas molecules reached the sensor housing. Therefore, as the concentration of the gas increased, the quantity of the adsorbed molecules on the surface of the sensing layer increased, and the electrons produced by that sensor also increased.

Moreover, the response of the gas sensor at the end of the simple microchannel was greater than in the serpentine model. As can be seen from the figure, different gases at different concentrations can have the same responses, as acetone gas at a concentration of 65 ppm has the same value response as ethanol gas at a concentration of 16 ppm in the simple model. Due to overlapping responses to acetone and ethanol gases in both sensor structures, it is not possible to discuss the selectivity of the gas sensor based on the variation in gas concentration. Therefore, it is more useful to use the normalized value of the gas response according to Equation (9). In the normalized response, the effect of concentration on the response value is ignorable. According to Equation (10), we defined a criterion for the discrimination of gas A and gas B. According to Equation (10), it is conceivable that if the difference between normalized currents of the sensor to various gases increases the selectivity of the sensor improves.

**Figure 9:**
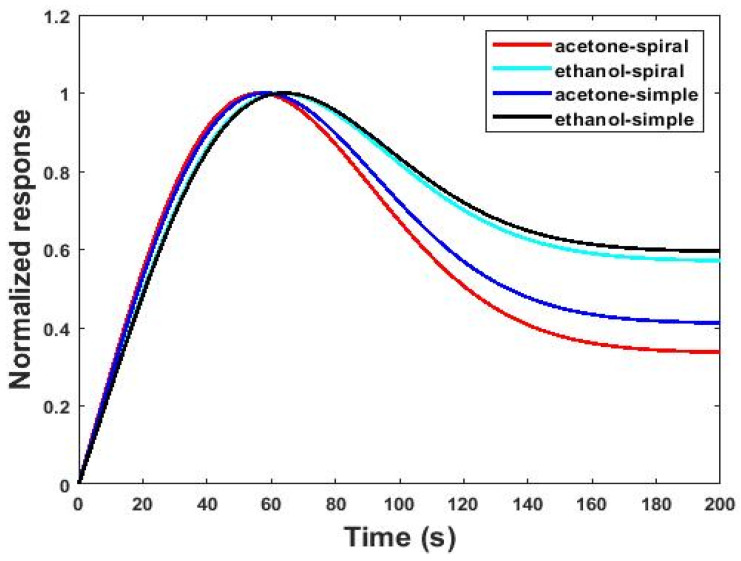
Selectivity of the sensor exposed to 2.6 ppm acetone and ethanol gases in both serpentine and simple models with the same dimensions at a 27 °C working temperature.

Generally, diffusion and adsorption/desorption mechanisms have impacts on the selectivity of the gas sensor, the same as the response [20,36]. Since dimensional channel variation affects the adsorption /desorption and diffusion mechanisms, these changes can also affect selectivity. The normalized responses for both the serpentine and simple models in the presence of 2.6 ppm acetone and ethanol gas at a 27 °C working temperature are shown in Figure 9. As can be seen from Figure 9, the differences in the normalized responses at cutting times (*t* ˃ 40 s) were obvious. The normalized response reported in Figure 9 is for channels with the same dimensions. The selectivity (S_AB_) was calculated from the response diagrams in Figure 9. S_AB_ was obtained for the serpentine channel sensor (221) and the simple channel sensor (141). These values show the selectivity of the gas sensor at the end of the serpentine microchannel was more than the simple model. As the channel became more serpentine, the collision of gas molecules with each other and the channel walls increased, and the diffusion rates and kinetic energy of the gas molecules that reached the sensor housing decreased. Therefore, most of the molecules tended to adsorb on the channel walls. Generally, various gases have different diffusion rates [45]. Therefore, increasing the serpentine aspect of the channel affects the collision of different gas molecules with each other and the channel walls as well as their loss of kinetic energy differently. Moreover, the concentrations of different gases that reach the sensor housing varies. Therefore, the ability of the sensor to identify and distinguish different gases from each other, and thus the selectivity of the sensor, increases.

Moreover, the length variation of the channel has a great impact on the diffusion rates of various gases. In fact, by increasing the length of the channel, the diffusion rates of molecules decrease due to the high probability of collision of the gas molecules with each other and the channel walls. All gases consist of molecules in constant random motion, but this constant random motion in different gas molecules varies according to their weights and structures [46]. Therefore, their speeds, accelerations and types of movement in different channels are different. On other hand, according to kinetic molecular theory, gas molecules do not stick to each other [47,48] and may only be adsorbed on the channel wall surface. Therefore, morphological changes are also effective channel adsorption mechanisms because molecules exert no other forces on each other. The deceleration and concentration of various gases reaching the sensor housing are different for various gases. Therefore, it can be concluded that by increasing the length of the channel the ability of the sensor to distinguish and separate different gases from each other increases. Therefore, the differences in normalized responses of the sensor to various gases increased and the selectivity (S_AB_) of the sensor improved.

**Figure 10:**
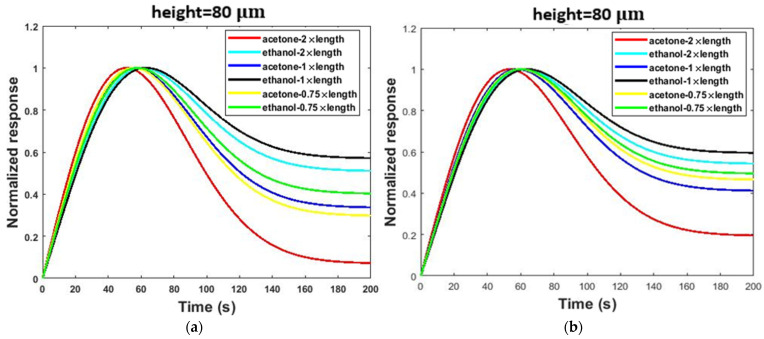
The effect of length variation of the channel on the selectivity of the sensor in the presence of 2.6 ppm acetone and ethanol gases in both the serpentine and simple models: (**a**) serpentine, (**b**) simple.

Figure 10 shows the effect of length variation of the channel on the selectivity of the sensor in the presence of 2.6 ppm acetone and ethanol gases. As can be seen from Figure 10, similarly, the greatest differences in normalized responses were seen at cutting times (*t* ˃ 40 s). The selectivity (S_AB_) was calculated from the normalized response diagrams in Figure 10. As presented in Table 2, S_AB_ increased by increasing the channel length. As shown in Table 2 and Figure 10, increasing the length from 22.5 to 60 mm led to a 77% increase in selectivity in the serpentine model and a 92% increase in selectivity in the simple model.

**Figure 11:**
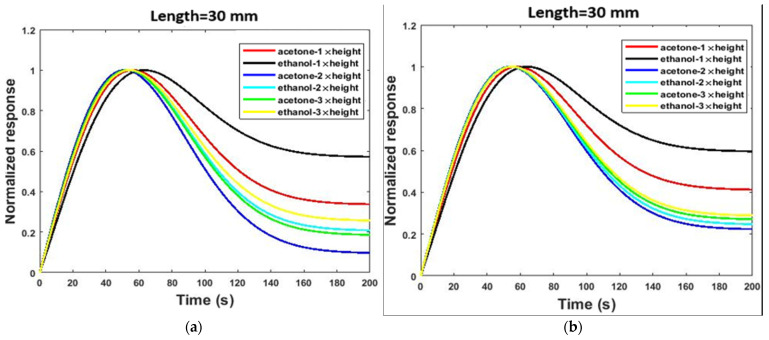
The effect of cross-section variation of the channel on the selectivity of the sensor in the presence of 2.6 ppm acetone and ethanol gases in both the serpentine and simple models: (**a**) serpentine, (**b**) simple.

As Figure 11 shows, channel cross-section variation affects selectivity the same as the length. Decreasing the channel height slows the diffusion and transfer rates of gas molecules and increases the effectiveness of physical adsorption. Thus, the selectivity (S_AB_) of the sensor improves. Figure 11 reaffirms that the greatest change in the normalized responses was evident during the cutting time. Moreover, selectivity (S_AB_) was calculated from the normalized response diagrams in Figure 11. The calculated results show S_AB_ increases by decreasing the height of the channel, as presented in Table 3. By increasing the length, the reduction in the cross-section area increases the collision of gas molecules with each other and the channel walls. As shown in Table 3 and Figure 11, decreasing the cross-section area from 240 to 80 µm led to a 67% increase in selectivity in the serpentine model and an 89% increase in selectivity in the simple model.

The gas exposure time was set in the simulation as less than the cutting time. This time estimation was based on trial and error to find the maximum selectivity. Generally, changes in the channel height and length have little effect on the exposure and cutting times. As a result, the gas exposure time was set at 40 s, and cutting time was about 160 s. As Figure 10 and Figure 11 and Table 2 and Table 3 show, the changes in the normalized responses during the cutting time were larger than the exposure time in all simulation results. This phenomenon may be due to the reduction in the kinetic energy of gases during the recovery process. In the presence of gas, the channel inlet was pumped with a certain concentration of gas. This constant concentration was defined as the driving force for the gas molecules and caused the uniform movement of molecules in the channel. Inlet gas was cut during the cutting time. Therefore, no external forces were applied to the gas molecules. In this case, the movement of gas molecules in the channel occurred randomly and depended only on environmental conditions, according to the kinetic theory of gases [44].

According to obtained simulation results, it can be concluded that the selectivity of the sensor depends only on the length, cross-section and shape of the channel and is independent of the gas concentration. As a result, the microchannel with dimensions of W = 3 mm, L = 60 mm and H = 80 μm has the highest and most optimal selectivity among the different geometries. The simulation results show the selectivity of the gas sensor in the presence of acetone and ethanol gases at the end of the serpentine microchannel was greater than in the simple model. In addition, the height and length variation of the serpentine microchannel had an effect on selectivity improvement. Reducing the channel surface area from an area of 0.72 mm^2^ to an area of 0.24 mm^2^ resulted in a 67% improvement in selectivity. Similarly, increasing the length from 22.5 mm to 60 mm increased the selectivity by 77% in the serpentine microchannel model. Generally, the most optimal dimensions of the serpentine microchannel, considering high sensitivity and selectivity, are W = 3 mm, L = 22.5 mm, and H = 80 µm.

**Figure 12:**
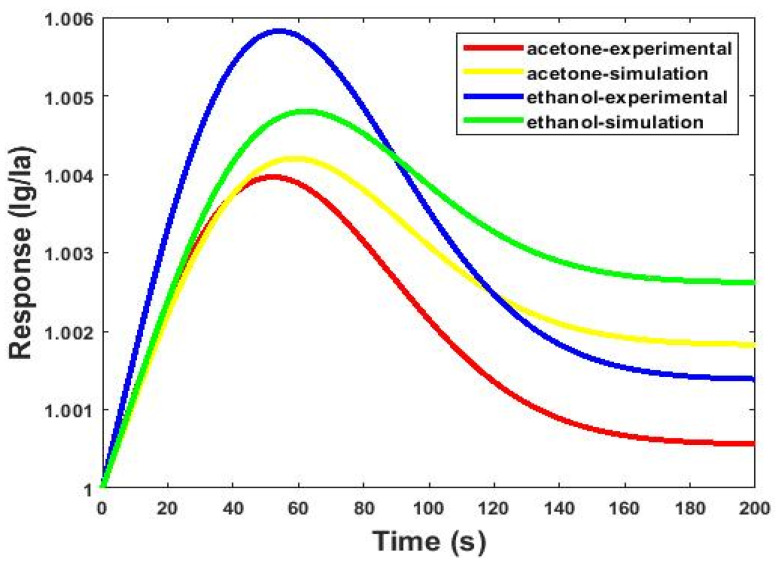
Comparison between the simulation and experimental results of the microfluidic-based gas sensor with optimal dimensions (W = 3 mm, L = 22.5 mm, H = 80 µm) in the presence of 2.6 ppm concentrations at 27 °C.

Obtaining equal results from an experiment and a simulation is a challenge for any research. The preliminary experimental tests of this study confirmed the simulation results. The experimental results were based on a real-time system, providing more accurate results compared to the simulation. The difference between the experimental and simulation results is there due to the errors that occurred from external disturbances, instruments, etc. Here, Table 4 and Figure 12 show a comparison of the simulation results and the real system. They show that the results obtained based on the simulated model are consistent with the fabricated system.

**Figure 13:**
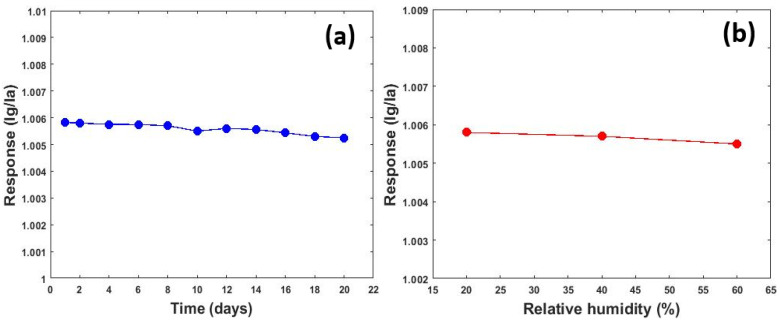
(**a**) Long-term stability of ZnO microfluidic-based gas sensor. (**b**) The effect of humidity on sensing response.

The stability of the ZnO microfluidic-based gas sensor was investigated for 20 days. The sensing responses were obtained for ethanol at room temperature almost every two days. Figure 13a shows the effect of the long-term stability of the ZnO microfluidic-based gas sensor. It can be understood that the sensing response had about 10% change in 20 days. Moreover, the effect of humidity on the microfluidic-based gas sensor is demonstrated in Figure 13b. While zinc oxide sensors without microfluidic channels are greatly affected by humidity variation [49], Figure 13b shows that the microfluidic channel can reduce the effect of humidity.

The results obtained from this research can lead to the development of microfluidic-based gas sensors by examining different channel structures. Moreover, the variety in channel structures can be an attractive idea for different gas-sensing applications to detect and discriminate mixed gases. Any mixtures of binary components have different diffusion coefficients. Therefore, gas identification from a mixture requires special patterns and modeling, which are investigated in the field of electronic noses.

Generally, the main aim of this work is different from previous works [1,10]. In this work, we simulated the gas sensors at the ends of two channels with different dimensions and designs (simple and serpentine). Then, we compared their sensing responses and selectivities to reach an optimal microchannel. However, we focused on the selectivity of the sensor to differentiate between two gases. Differentiating between polar and nonpolar gases in much easier than differentiating between two polar or two nonpolar gases. In this work, we considered two polar gases (ethanol and acetone) and investigated their selectivity. We gave a specific definition for selectivity as Equation (10) and compared the selectivity of the target gases according to this equation.

## 5. Conclusions

This study presented a simulation of a PDMS microfluidic-based gas sensor in both simple and serpentine models of the channel. The simulation results helped to understand some parameters affecting the gas-sensing mechanism of the sensor. Among them, diffusion and adsorption phenomena have many impacts on the gas response and selectivity. To reach optimal channel geometry, the simulation was used to investigate dimensional effects on selectivity and the responses of the sensors. It was found that the sensor with a simple channel has more gas response and less selectivity than the sensor with the serpentine channel. Generally, as the cross-section of the channel increases and its length decreases, the gas response of the sensor increases. Moreover, the selectivity in both models of sensors increases dramatically with increasing channel length and decreasing cross-sectional area. According to performed simulations, a microchannel with dimensions of W = 3 mm, H = 80 µm and L = 22.5 mm is the optimal geometry with high selectivity and gas response.

## Figures and Tables

**Table 1:** Different dimensions of microchannel geometry for simulation.

Simulation Round	Channel Width	Channel Height	Channel Length
1	W	1 × H	0.75 × L
2 ^1^	W	1 × H	1 × L
3	W	1 × H	2 × L
4	W	2 × H	0.75 × L
5	W	2 × H	1 × L
6	W	2 × H	2 × L
7	W	3 × H	0.75 × L
8	W	3 × H	1 × L
9	W	3 × H	2 × L

^1^ Reference dimensions: Initial dimensions of the channel.

**Table 2:** Comparison of calculated selectivity (S_AB_) by changing the channel length of the sensor in the presence of 2.6 ppm acetone and ethanol gases in both the serpentine and simple models.

Dimensions			Simple	Serpentine
W	H	L	S_AB_	S_AB_
3 mm	80 µm	60 mm	337	448
3 mm	80 µm	30 mm	141	221
3 mm	80 µm	22.5 mm	28	103

**Table 3:** Comparison of calculated selectivity (S_AB_) by changing channel height of the sensor in the presence of 2.6 ppm acetone and ethanol gases in both the serpentine and simple models.

Dimensions			Simple	Serpentine
W	L	H	S_AB_	S_AB_
3 mm	30 mm	80 µm	141	221
3 mm	30 mm	160 µm	24	114
3 mm	30 mm	240 µm	16	72

**Table 4:** Comparison between simulation and experimental results of the microfluidic-based gas sensor with optimal dimensions (W = 3 mm, L = 22.5 mm, H = 80 µm) in the presence of 2.6 ppm concentrations at 27 °C.

Responses and Selectivity Indicators of the Microfluidic-Based Gas Sensor			
	Response to Acetone	Response to Ethanol	S_AB_
Simulation	1.0042	1.0048	103
Experimental	1.0039	1.0058	98

## Data Availability

The data presented in this study are available from the corresponding author upon reasonable request.
